# Exploring the Therapeutic Potential of Medicinal Plants in the Context of Gastrointestinal Health: A Review

**DOI:** 10.3390/plants14050642

**Published:** 2025-02-20

**Authors:** Antonio Julián-Flores, Pedro Aguilar-Zárate, Mariela R. Michel, Leonardo Sepúlveda-Torre, Cristian Torres-León, Cristóbal N. Aguilar, Mónica L. Chávez-González

**Affiliations:** 1 Bioprocesses & Bioproducts Group, Food Research Department, School of Chemistry, Autonomous University of Coahuila, Saltillo 25280, Coahuila, Mexico; antoniojulian@uadec.edu.mx (A.J.-F.); leonardo_sepulveda@uadec.edu.mx (L.S.-T.); cristobal.aguilar@uadec.edu.mx (C.N.A.); 2 Laboratorio Nacional CONAHCYT de Apoyo a la Evaluación de Productos Bióticos (LaNAEPBi), Unidad de Servicio, Tecnológico Nacional de México/I.T. de Ciudad Valles, Ciudad Valles 79010, San Luis Potosí, Mexico; mariela.michel@tecvalles.mx; 3 Research Center and Ethnobiological Garden, Autonomous University of Coahuila, Viesca 27480, Coahuila, Mexico; ctorresleon@uadec.edu.mx

**Keywords:** gastrointestinal diseases, intestinal microbiota, medicinal plants, bioactive compounds

## Abstract

Medicinal plants represent promising sources for the treatment of gastrointestinal disorders because of their abundance in bioactive compounds with therapeutic properties. Throughout history, various plant species have been used to alleviate digestive ailments, and studies have revealed the presence of metabolites with anti-inflammatory, antibacterial, antiviral, antiparasitic, antidiarrheal, antioxidant, and anticancer activities. The secondary metabolites responsible for these properties include alkaloids, terpenoids, and phenolic compounds, with the latter, particularly flavonoids, being the most associated with their bioactivities. Gastrointestinal diseases, such as gastritis, peptic ulcers, gastroesophageal reflux disease, inflammatory bowel disease, irritable bowel syndrome, and gastrointestinal cancer, are caused primarily by bacteria, parasites, viruses, and the consumption of raw or undercooked foods. These conditions significantly impact human health, necessitating the development of safer and more effective therapeutic alternatives. After an extensive literature review, several plant species with widespread use in the treatment of these disorders were identified, including *Matricaria chamomilla, Mentha spicata, Melissa officinalis, Artemisia ludoviciana, Flourensia cernua, Phoradendron californicum*, and *Turnera difusa*. This study revealed that the analyzed plants are rich in bioactive compounds, which confer their medicinal properties. However, many other plants commonly used to treat digestive disorders have been scarcely studied, highlighting the need for further research.

## 1. Introduction

Gastrointestinal diseases (GDs) pose a significant public health challenge worldwide, affecting millions of people. GDs are disorders that affect the esophagus, stomach, intestines, rectum, and other digestion-related organs [1]. Affected individuals experience significant symptoms such as constipation, diarrhea, abdominal pain, and alternating constipation/diarrhea [2]. These conditions are partly related to the intestinal microbiota (IM), which maintains a relationship with its host in the gastrointestinal tract [3]. Several studies suggest that plant-based diets may be helpful in the treatment and prevention of most gastrointestinal tract diseases [4,5]. Since ancient times, humans have used plants to treat infectious diseases. Scientific research has demonstrated the therapeutic efficacy of plants over time. Today, many countries use medicinal plants to treat different diseases, including infectious diseases of the respiratory, gastrointestinal, urinary, and biliary systems [6]. The secondary metabolites present in medicinal plants, such as polyphenols, terpenoids, and alkaloids, exhibit biological properties that have the potential to benefit human health [7]. These compounds have been used to identify new drugs or phytomedicines [8]. However, not all secondary metabolites provide health benefits; some can be toxic to humans when present in high concentrations [9]. There are interactions between the IM and drugs in the gastrointestinal tract, where changes in the composition of the IM can occur and at the same time, can cause chemical transformations in the drugs themselves [10]. Therefore, the generation of new drugs is essential since the excessive use of antibiotics has become a global problem due to the generation of microbial resistance [11]. The objective of this review is to present several traditional plants that help counteract GDs and their associated bioactive compounds, which may be used as potential ingredients in new drugs.

## 2. Research Methodology

In this review article, a comprehensive search was performed in the following databases: ResearchGate, ScienceDirect, SpringerLink, Scopus, Wiley Online, Semantic Scholar, and Google Scholar. The key words used for the information search were “medicinal plants”, “gastrointestinal diseases”, “intestinal microbiota”, “bioactive compounds”, and “medical failure”. The inclusion criteria encompassed review articles, research findings, books, and book chapters, studies conducted in Spanish and English, and plants from any region of the world. The exclusion criteria include articles without full-text access, duplicate studies, and thesis works.

## 3. Gastrointestinal Diseases

Gastrointestinal diseases are the complex illnesses of various parts of the gastrointestinal tract, which affect the esophagus, stomach, small intestine, large intestine (colon), and rectum [12]. These problems can range from mild discomfort to serious conditions and may involve symptoms such as abdominal pain, bloating, gas, nausea, vomiting, diarrhea, constipation, heartburn, and rectal bleeding, among others [13]. Reports from the World Health Organization (WHO) quantify the burden of disease via disability-adjusted life years (DALYs). This means calculating the number of healthy life years lost due to illness and death to facilitate the classification of global and regional disease causes. In 2019, digestive diseases accounted for an estimated 2276.27 million prevalent cases, with 2.56 million deaths and 88.99 million DALYs, which were primarily concentrated in countries in Africa, the Middle East, and Central Asia. They represent a global issue, with the number of incident cases of digestive diseases worldwide reaching 443.53 million [14]. In addition, gastrointestinal conditions entail the significant use of healthcare and financial resources. In the United States alone, in 2018, $119.6 billion was allocated to gastrointestinal healthcare expenditures. These diseases impact patients’ physical health and can significantly affect their quality of life and their ability to perform daily activities [15].

In Table 1, several of the most important diseases that significantly affect the digestive tract in the global population are presented:

## 4. Microbiota and Gastrointestinal Health

**Figure 1:**
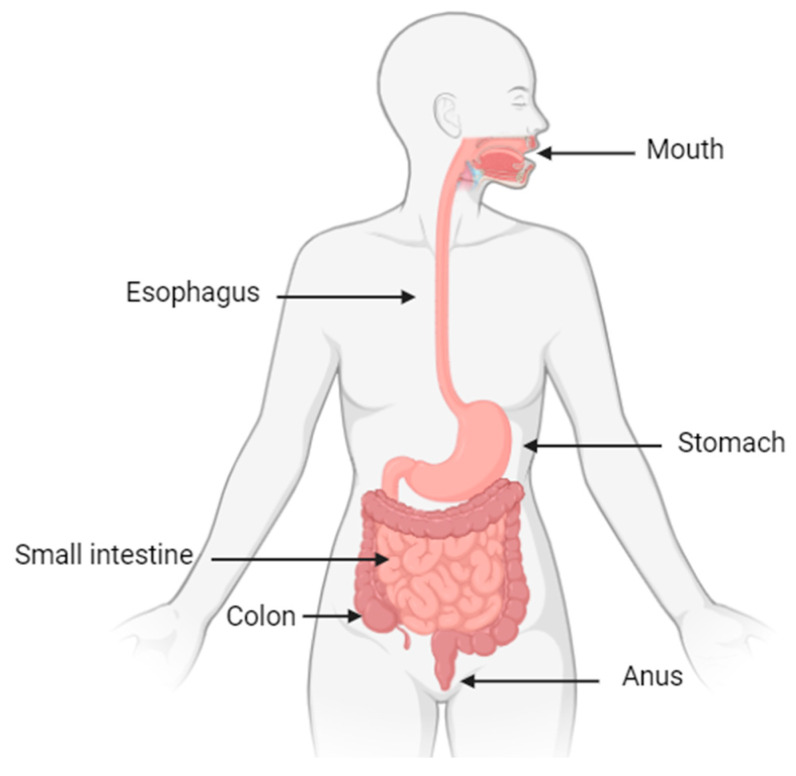
Parts of the digestive system. Own source.

The digestive system plays a fundamental role in the overall well-being of the human organism. This system is composed of complex organs, beginning in the mouth and continuing through the esophagus, stomach, small intestine, large intestine, and finally the anus (Figure 1). The small intestine is where most digestion occurs and is responsible for nearly all nutrient absorption [29].

The IM plays a very important role in the digestive tract, providing numerous benefits to the host organism through various physiological functions, including strengthening the integrity of the gut, contributing to the formation of the intestinal lining, providing energy, protecting against pathogens, and regulating the host immune response [30]. The IM is composed of various species of microorganisms, such as bacteria, yeasts, and viruses, including *Firmicutes, Bacteroidetes, Actinobacteria, Proteobacteria, Fusobacteria*, and *Verrucomicrobia*, with the first two representing 90% of the IM [31]. Additionally, it is an important factor in contributing to regulation of the intestinal mucosal barrier function, which is essential because it is a complex barrier that must exclude bacteria and their molecular toxins while absorbing vital nutrients for homeostasis. Moreover, it stimulates hosts to produce antimicrobial compounds to protect against pathogenic agents [29,32].

Several factors affect the well-being of the intestinal tract. The composition of the host diet affects both the structure and metabolism of the IM, as microorganisms are closely related to diet and different physiological states. This is due to their ability to produce intestinal microbial metabolites in response to dietary intake [33]. The consumption of fiber-rich foods is associated with a decrease in the frequency and severity of various diseases, including colon, breast, and liver cancers, cardiovascular diseases, respiratory infections, diabetes, and obesity [34]. Additionally, the intake of prebiotics, in the form of foods or medications, promotes stability in the digestive system because they serve as a substrate for the nourishment (known as “microbiota-accessible carbohydrates”) of the microorganisms present in the intestine. Similarly, when probiotics are administered appropriately, their consumption confers health benefits to the host [35].

Some researchers report the significant influence of stress on the connection between the brain and the intestine, which generates important consequences for several common diseases that manifest with chronic gastrointestinal symptoms [36]. The brain can influence the composition and function of the IM through the autonomic nervous system, controlling intestinal motility, transit, secretion, and permeability [37].

Increasing evidence suggests that these factors shape the intestinal microbiome throughout an individual’s life, generating a unique and specific microbial composition for each person [38]. Therefore, to prevent or mitigate potential gastrointestinal diseases, maintaining a healthy diet rich in fiber and incorporating foods with high probiotic and prebiotic contents are necessary to promote good digestive health.

## 5. Insufficiency of Medical Services and Backwardness

The World Health Organization has mentioned that each year, millions of patients experience harm because of unsafe healthcare, resulting in 2.6 million annual deaths in low- and middle-income countries alone. The right to universal health coverage (UHC) law exists. However, the financial resources of each country differ, so developing countries struggle to provide high-quality healthcare services. The WHO global health statistics (2020) indicate that approximately 33% to 49% of the world’s population had access to essential health services in 2017. More than 40% of countries have fewer than ten doctors per 10,000 people, and more than 55% have fewer than 40 nurses and midwives per 10,000 people.

According to the National Institute of Statistics and Geography (INEGI), in the year 2020, out of the 126,014,024 people in Mexico, 32,999,713 were not affiliated with any health service, meaning that 26.2% of the population did not have access to a public health institution. Therefore, some people turned to alternative medicines, including medicinal plants. When surveyed, 44.8% used traditional medicine products, and 55.2% of the population resorted to traditional medicine services, commonly known as “herbalists”.

Medicinal herbs have been used and accepted by the general population worldwide. Several medications are used to treat GDs, such as sucralfate’ H_2_ receptor (histamine) antagonists in clinical use, such as cimetidine, ranitidine, famotidine, and nizatidine; antacids; antibiotics; and proton pump inhibitors (PPIs), including the most common types in clinical practice: omeprazole, esomeprazole, lansoprazole, dexlansoprazole, rabeprazole, and pantoprazole [39]. Although there are a variety of drugs that help counteract GDs, the high cost makes their accessibility difficult for low-income sectors.

## 6. Traditional Uses of Plants

Plants represent the primary sources of essential food for the existence and survival of humans, animals, and microorganisms. Over time, humans have acquired knowledge about the use of plants in food preparation and medicine through practical experimentation, allowing them to gradually meet their needs using the resources available in their environment [40]. Empirical wisdom about the positive effects of plants has been passed down from generation to generation within human communities [41].

Medicinal plants are widely used in traditional cultures worldwide and are gaining popularity in modern society as natural alternatives or complements to synthetic chemicals [42]. Most aromatic and medicinal plants are found in the regions with the richest biodiversity in the world, but with less developed economies. Therefore, the collection and trade of these plants significantly contributes to the livelihoods of communities and local economic development [43]. Plants are recognized as reservoirs of a wide variety of bioactive compounds and have been employed extensively due to their healing properties [44]. Medicinal plants play essential roles in curing most diseases, as many human disorders are treated with medications derived from plant components [45].

**Figure 2:**
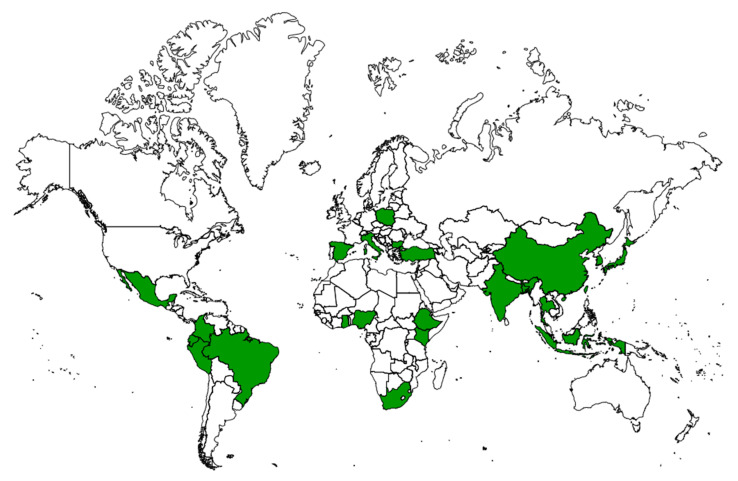
Countries with a greater use of medicinal plants. Own source.

According to the WHO, more than 80% of the global population more frequently resort to traditional plant-based medicines. One hundred and seventy WHO members states from a total of one hundred and ninety-four reported the use of medicinal herbs and other forms of traditional medicine. Traditional medicinal systems in Asia have been practiced since ancient times. Notable examples include Jamu (Indonesia), Traditional Chinese Medicine (TCM) (China), Kampo (Japan), and Thai medicine (Thailand). On the other hand, nations such as Korea, India, Malaysia, and Vietnam have registered and published national monographs on plant-based drugs [46]. The main producers of medicinal and aromatic plants in Europe are Bulgaria (20.2%), Spain (19.4%), Italy (8.4%), Poland (7.0%), and Turkey (10.0%) [47]. The African continent stands out for the abundance of medicinal plants in South Africa (3000 plants) and thousands more in Egypt, Morocco, and Algeria in the north; Nigeria and Ghana in the west; Cameroon and Gabon in the center; and Kenya and Tanzania in the east [48]. In terms of the number of medicinal plants, North America is highlighted by the United States and Mexico, whereas South America has high biodiversity in Brazil, Chile, Guyana, Peru, Bolivia, Argentina, and Venezuela [42]. Asian medicinal plants represent approximately 50% of exports and 45% of global revenue derived from traditional medicine, led by China and India [46]. Figure 2 shows the countries that use the greatest number of plants per continent (with the exception of Oceania).

Medicinal herbalism plays a fundamental role in Mexico, as evidenced by its presence in contemporary Mexican markets where large quantities of medicinal and aromatic plants are commercialized, reaffirming its marked effectiveness against various diseases in the population [49]. In Mexico, medicinal plants are the primary alternative for treating diseases among economically vulnerable populations. The Mexican health secretary mentioned that in 2021, Mexico possessed one of the most extensive regions of botanical diversity worldwide. Therefore, 90% of the Mexican population has opted for the use of at least one of the 4500 medicinal plants of Mexico at least once in their life. Depending on the need or specific prescription, different parts of the plant are employed, with the leaves or flowers being the most common, although the stem and root are occasionally used. These parts are consumed directly, commonly through infusions or other homeopathic preparations [50]. The various parts of the plant (leaves, roots, bark, fruits, or seeds) generally contain distinct active principles from each other, which may imply that one part is toxic whereas the other is safe for use [42].

## 7. Plants That Improve Gastrointestinal Health

To address digestive system issues, alternatives in herbal medicine have been sought, as they represent a valuable source of essential medicines and products based on medicinal plants for health care and disease treatment [51].

Below, different plants reported by rural communities and the general population are briefly described, some of which are poorly known but have potential benefits for gastrointestinal health.

### 7.1. Matricaria chamomilla

The plant *Matricaria chamomilla* from the Asteraceae family is distributed worldwide and has been used throughout history in traditional medicine to improve digestion, alleviate headaches and toothaches, and act as a sedative agent. [52]. The plant contains various active chemical components of great therapeutic value, primarily its flavonoids, such as apigenin, luteolin, and quercetin, as well as its sesquiterpenes, mainly β-farnesene, chamazulene, and α-bisabolol. In addition, it contains coumarins (herniarin and umbelliferone) [53]. These compounds are attributed to their biological activities such as antimicrobial, antioxidant, anti-inflammatory, antiulcer, hypoglycemic, cardioprotective, hepatoprotective, neuroprotective, antidiarrheal, wound healing, and anticancer properties [54]. This herb has fine, spindle-shaped roots. The stem reaches a height of 10–80 cm. The leaves are narrow and long, with golden-yellow flowers and floral heads with a diameter of 10–30 mm. The fruit is a brownish-yellow cypsela [55].

### 7.2. Mentha spicata

This species of green mint of the Lamiaceae family is widely distributed throughout the world. It is highly valued on the market for its aromatic and medicinal properties and is used in the food industry and in various industrial applications [56]. Its leaves are considered beneficial for strengthening the stomach and relieving symptoms associated with dyspepsia, such as the loss of appetite, bloating, belching, indigestion, and nausea [57]. Several active phytochemical compounds belonging to the different categories of secondary metabolites have been identified, particularly essential oils from the aerial parts, such as limonene, carvone, pulegone, and 1,8-cineole. Additionally, flavonoids, including apigenin, epicatechin, and myricetin, as well as phenolic acids such as caftaric acid, caffeic acid, and chlorogenic acid, have been documented [58]. It is associated with a wide range of biological effects, including antibacterial, antioxidant, hepatoprotective, antidiabetic, cytotoxic, anti-inflammatory, antigenotoxic, and antiandrogenic properties [59]. *Mentha spicata* is a perennial herb that spreads through long, slender rhizomes, reaching heights of 10 to 120 cm. Its leaves are broad, ovate, or lanceolate at the base, pubescent, and have thick veins [60].

### 7.3. Melissa officinalis

The *Melissa officinalis* plant is a perennial aromatic plant that is commonly found in the Mediterranean region and Western Asia and is widely cultivated in Europe [61]. It belongs to the Lamiaceae family and is used as a sedative and, owing to its digestive properties, to treat nervous anxiety, depression, tension headaches, and indigestion. In addition, it is used to treat diseases related to the liver and gallbladder [62]. It has anti-inflammatory, antimicrobial, antioxidant, sedative, and neuroprotective properties [63]. Its composition includes flavonoids such as quercitrin, rhamnocitrin, and luteolin, as well as polyphenolic compounds. Additionally, it contains phenolic compounds and essential oils (such as monoterpenoid aldehydes, monoterpene glycosides, triterpenes, and sesquiterpenes) [64]. It is a shrub-like plant that reaches heights of 60 to 100 cm. It has soft and velvety-textured leaves, measuring 2 to 8 cm in length, with a heart shape and a dark green color [61].

### 7.4. Artemisia ludoviciana

The genus *Artemisia* of the Asteraceae family is widely distributed in North America, mainly in the United States and Mexico [65]. It has antibacterial, antiviral, antiparasitic, antidiarrheal, and antifungal properties, as well as nematicidal and insecticidal effects [66]. Several researchers have employed extracts of *Artemisia ludoviciana* for the treatment of digestive, hepatic, and biliary diseases [67], as well as for treating respiratory conditions, in addition to their use as an antiparasitic agent in humans [68]. Phytochemical research on *A. ludoviciana* has led to the isolation of monoterpenoids (camphor and limonene), sesquiterpene lactones (estafiatin and ludovicin), and flavonoids (eupatilin and jaceosidin) [69]. It is also known as white sagebrush or silver wormwood because its shades range from grayish-green to silvery-white. It is a perennial herb that reaches a height of 30–70 cm. Its alternate leaves can be entire or lobed, measuring between 3 and 11 cm in length and up to 1.5 cm in width, and are covered by a dense layer of short, tangled hairs [65].

### 7.5. Flourensia cernua

The shrub *Flourensia cernua* of the Asteraceae family is an endemic species that grows in semiarid areas and is found in the deserts of Mexico and the United States. It has been the subject of numerous studies, as it has demonstrated antifungal, antibacterial, and antioxidant properties [70]. In the genus *Flourensia*, chemical compounds with significant biological activity have been discovered, primarily flavonoids, including chrysin, galangin, apigenin, kaempferol, quercetin derivatives, flavanones, 8-prenyl-flavanones, 8-prenyl-flavonols, and 5-acetylbenzofurans, as well as sesquiterpenes [71]. Owing to its composition of various bioactive agents, this plant has been used to treat different gastrointestinal conditions, such as stomach pain, diarrhea, and dysentery, and it has also been used as a purgative agent in the form of an infusion [72]. This genus comprises 40 species of aromatic and resinous shrubs, distinguished by the presence of an oily layer on the surface of their leaves and the abundance of yellow-colored flowers [71].

### 7.6. Phoradendron californicum

The species *Phoradendron californicum* is a hemiparasitic plant that grows on the stems and branches of its host plants. It is distributed in the southwestern United States and northern Mexico. Although it performs photosynthesis to produce its own carbohydrates, it also extracts water and nutrients from the xylem of its host, causing a negative impact on it [73]. In arid environments, typical hosts are green stick (*Cercidium* sp.) and mesquite (*Prosopis* sp.). The leaves and bark of this plant are used to relieve stomach discomfort and digestive problems, and are combined with other herbs in infusions to treat venereal diseases [74]. The secondary metabolites found in this species are influenced by various factors, such as the phenolic compounds of the host tree, nitrogen-related stress, the number of parasitic plants affecting the host tree, and other factors derived from biological interactions. Some of the identified metabolites include phenolic acids and flavonoids such as gallic acid, catechin, rutin, quercetin, and esculetin [73]. The perennial shrubs of the *Phoradendron* family of Viscaceae typically have stems that are greater than 25 cm in height. The leaves can be foliaceous or reduced to connect the deltoid scales, and the plants may be hairless or pubescent. The inflorescences, which are found in the leaf axils, are articulated or occasionally terminate in spikes [75].

### 7.7. Turnera diffusa

**Figure 3:**
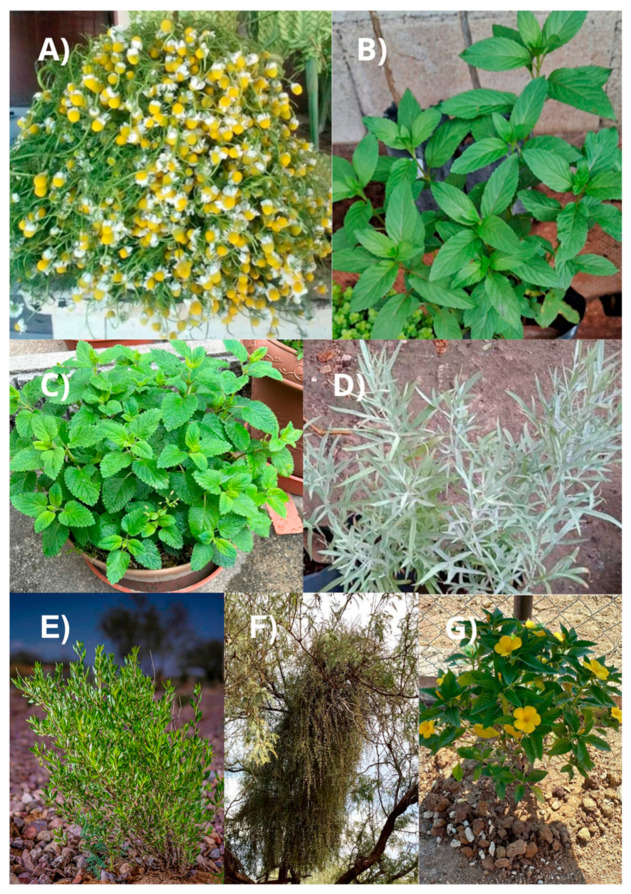
Medicinal plants promoting gastrointestinal health: (**A**) *Matricaria chamomilla*; (**B**) *Mentha spicata*; (**C**) *Melissa officinalis*; (**D**) *Artemisia ludoviciana*; (**E**) *Flourensia cernua*; (**F**) *Phoradendron californicum*; (**G**) *Turnera diffusa*. Own source.

The spearmint *Turnera diffusa* of the Turneraceae family is present on the American continent in arid and semiarid areas of the West Indies, South America, Mexico, and the United States [76]. It is traditionally used for the treatment of various ailments, such as sexual impotence, neurasthenia, diabetes mellitus, urinary retention, malaria, diarrhea, and peptic ulcers [77]. Flavonoids play crucial roles as the metabolites present in these plants, as their medicinal properties are attributed to them [78]. Thus, this plant has been associated with multiple ethnopharmacological uses, one of which is hepatoprotective because it contains a flavonoid called “hepatodamianol” which helps counteract liver problems. *T. diffusa* is also recognized for its activation of the nervous system and its aphrodisiac, diuretic, hypoglycemic, and antimicrobial properties [79]. Morphologically, it is a small, branched shrub that reaches heights between 60 cm and 1 m, with lanceolate leaves ranging from 10 to 25 mm in length. It produces small, round, fragrant fruits along with yellow flowers that bloom in the summer [80]. Figure 3 shows the different plants discussed above.

## 8. Phytochemicals as Treatments for Gastrointestinal Disorders

Throughout history, natural resources have been utilized for the benefit of health, with people employing natural reserves such as plants, animals, microorganisms, and marine organisms to manufacture medicines to mitigate and combat diseases [81]. It is known that vegetation is the primary source for medicinal production because of its biological properties. In recent years, there has been an increase in the acceptance of medicinal plants, driven by the belief that natural products have fewer side effects and are more effective than their synthetic counterparts [82]. Herbal medicines may include purified plant derivatives, crude extracts, specific formulations, and other preparations. At times, the concept of herbal remedies can be expanded to include medicinal materials derived from fungi, minerals, and animals [83]. Generally, plant-derived drugs are categorized on the basis of their source, composition, taste, and effectiveness [84].

**Figure 4:**
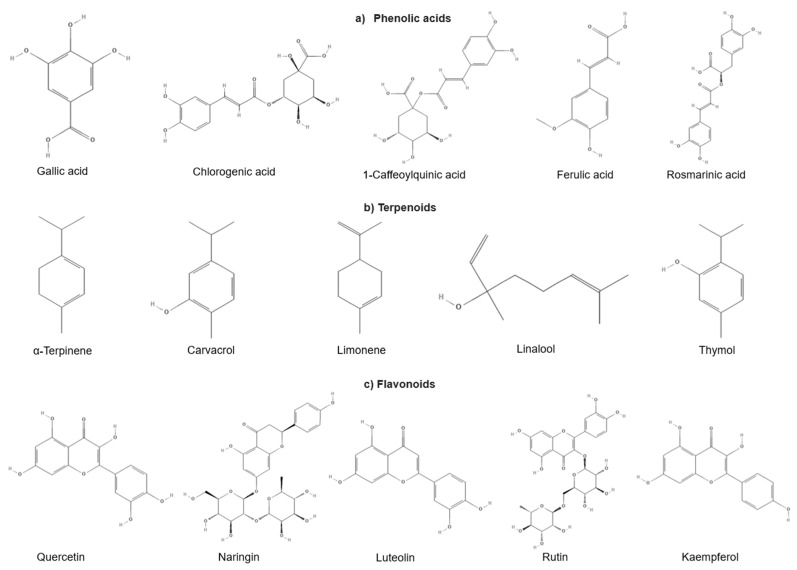
Chemical structures of the main molecules associated with gastrointestinal diseases. Own source.

The compounds responsible for the properties of plants are known as secondary metabolites that protect plants against various types of stress, whether of a biotic or abiotic origin, such as infections, threats from predators, exposure to ultraviolet radiation, and stress situations due to a lack of water or salinity [85]. The health benefits of plants are due to the immense diversity of specialized metabolites such as alkaloids, terpenoids, or phenolic compounds, which confer diverse physiological effects on the human body [45]. They possess various properties such as anti-inflammatory, antioxidant, antiviral, antifungal, antibacterial, and antitumor effects [86]. Figure 4 illustrates the chemical structures of the main phytochemicals associated with gastrointestinal disorders. However, secondary metabolites vary depending on the growing season, years of development, and environment. The quantity of these metabolites may increase or decrease during the growth process or in stressful situations, even in medicinal plants with identical genetic backgrounds [87]. Table 2 shows the plants reported to have potential benefits to the digestive system and the metabolites associated with their properties.

More than 50% of the new drugs developed and authorized for sale originate directly from the altered compounds of medicinal plants or the active compounds found in these plants [103]. The discovery of medications from natural resources, considering ethnopharmacological analyses, plays a significant role in advancing contemporary medical treatments [104]. Industrialized nations heavily rely, albeit indirectly, on medicinal plants to produce their medications. It is estimated that 25% of the world’s modern pharmacopeia is of plant origin [46].

In 2022, the WHO made efforts in traditional medicine by establishing the Global Traditional Medicine Center (GTMC) to integrate ancient knowledge and modern science. Additionally, it created the International Pharmacopoeia, which is an official compendium containing a list of drugs and their formulas, as well as standards for their preparation and use. Its concept originated from the ancient Greek term ϕαρμακοποιΐα (pharmakopoiia), which derives from ϕάρμακον (pharmakon) meaning “drug”, along with the verb ποι- (poi-) meaning “to make”, and finally the abstract noun ending -ια (-ia). The combination of these components can be interpreted as “drug making” [105].

In Mexico, the Pharmacopeia of Herbal Medicine of the United Mexican States (FHEUM), issued by the Secretariat of Health, sets out the general methods of analysis and the technical specifications of the plants and their derivatives used in the preparation of medicines and herbal remedies. There are different editions of this document that provide information about medicines to ensure the quality of products in the market [106]. Plant therapies are traditionally used in multiple pharmacopoeias and across a wide range of doses, including highly diluted homeopathic formulations [107].

The collection of plants is a frequent activity in rural communities, where the collected plants are used to treat different ailments or diseases. They are mainly used for consumption in infusions and decoctions, using different parts of the plant, as appropriate. However, there is no control over the collection of plant material or its geolocation, which may affect the bioactive compounds and consequently reduce the effectiveness of its bioactive properties. In addition, there are no practices for using and exploiting these plants, which could result in their extinction.

## 9. Toxicity of Plants In Vitro and In Vivo

It has been demonstrated that the aqueous extracts of *M. chamomilla* exhibit toxicity, as evidenced by the inhibition of bioluminescence in the Gram negative marine bacteria *Vibrio fischeri*, with concentrations ranging between 0.032 and 1.264 mg/mL. The compounds obtained through infusion extraction included rutin trihydrate, ferulic acid, chlorogenic acid, and apigenin-7-O-glucoside [108]. On the other hand, the essential oil of *Mentha spicata*, which contains carvone, limonene, and dihydrocarvone, has been shown to be toxic to the insect *Reticulitermes dabieshanensis*. Among its main components, carvone, dihydrocarvone, and limonene were the most effective against *R. dabieshanensis*, with values of 0.074, 0.155, and 2.650 μL/L, respectively [109]. A toxicity assessment of *M. officinalis* was conducted on the essential oils extracted from the aerial parts through hydrodistillation. When administered orally to mice at doses exceeding 1 g/kg, various pathological changes were observed in the stomach, duodenum, liver, and kidneys [110]. To evaluate the cytotoxicity of the ethanolic extracts of *A. ludoviciana*, an ex vivo assay was conducted using the human macrophage line THP-1, along with an in vivo assay using the *Artemia* species. In the first model, the extract concentrations ranging from 5 to 20 μg/mL were tested, with cytotoxic effects observed at concentrations of 20 μg/mL or higher. In the *Artemia* model, the ethanolic extract was evaluated within a concentration range of 10–1000 μg/mL. Following exposure of the nauplii, moderate toxicity was observed at 195.64 μg/mL [111]. For the shrub *F. cernua*, the cytotoxic and antiproliferative activities of its ethanolic extracts obtained via Soxhlet extraction were evaluated in A549 and ARPE-19 cells at concentrations of 0, 6.25, 12.5, 25, and 50 μg/mL. The results demonstrated that in A549 cells, a concentration of 12.5 μg/mL resulted in a cell viability of 87.9%, whereas in ARPE-19 cells, a viability of 50.76% was observed at a concentration of 25 μg/mL [112]. The cytotoxic effects of the hemiparasitic plant *P. californicum*, were evaluated in the HeLa and PC3 cancer cell lines using methanolic and chloroform extracts obtained through maceration for three days at a 1:10 ratio. The results indicated that the chloroform extract exhibited greater antiproliferative activity in the tested cell lines (215.62 ± 14.70 µg/mL and 167.67 ± 5.08 µg/mL, respectively) than the methanolic extract (340 ± 11.58 µg/mL and 352.51 ± 9.87 µg/mL) [113]. Additionally, *T. diffusa* has been shown to act as a modulator in reducing testicular toxicity levels in rats induced by fenitrothion exposure. Furthermore, it has the potential to increase the levels of the hormones, including testosterone and follicle-stimulating hormone, in rats [114].

## 10. Future Perspectives and Conclusions

Despite the significant progress made in the chemical characterization of the species used in traditional medicine, much remains to be done. There are still hundreds of species for which the active chemical compounds related to their attributed beneficial effects are unknown. In recent years, many research groups have been trying to identify compounds with biological activity to contribute to greater knowledge. With a combination of scientific research, clinical studies, treatment customization, and community collaboration, it is possible to harness the therapeutic potential of medicinal plants safely and effectively to improve gastrointestinal health and overall well-being.

Maintaining good gastrointestinal health is very important, as it affects digestion and nutrient absorption and influences immune function, mood, metabolism, and disease prevention. For centuries, medicinal plants have been used in various cultures as natural remedies to promote gastrointestinal health and treat a wide range of digestive disorders. Over time, scientific research has supported many of these traditional practices, identifying plant bioactive compounds that benefit the gastrointestinal tract. These natural compounds can provide safe and effective alternatives to synthetic medications, with fewer side effects and broader potential for treatment customization. On the other hand, it is essential to mention that the use of herbal medicine is partly due to the high costs of medications, which are often economically inaccessible to vulnerable communities. Therefore, generating knowledge and providing livelihoods, economic development, and scientific advancement to the communities where these plant species are found is essential.

Knowing and conserving medicinal plants are vital for several reasons; their traditional knowledge and use represent the intelligence and wisdom of societies/communities accumulated over time, which must be preserved and passed on to future generations. Additionally, ensuring their continued availability and long-term sustainability is crucial.

## Figures and Tables

**Table 1:** Characteristics of gastrointestinal diseases.

Gastrointestinal Diseases	Description	Etiologic Agent	Common Symptoms	References
Gastritis	Inflammation of the gastric mucosa	- *Helicobacter pylori* infections - Stress - Excessive use of non-steroidal anti-inflammatory drugs (NSAIDs) - Alcohol consumption	Stomach pain, abdominal distension, nausea, vomiting, and loss of appetite	[16,17]
Peptic ulcer	Lesion in the digestive tract caused by acid, which is usually found in the stomach or proximal duodenum	- *Helicobacter pylori* infections - Inappropriate diet - Alcohol and tobacco - NSAIDs	They are nonspecific, but some present with postprandial abdominal pain, nausea, vomiting, and weight loss	[18,19]
Gastroesophageal reflux disease	Occurs when stomach contents flow back into the esophagus, causing a series of complications and discomfort	- Obesity - Smoking - Genetic predisposition	Include heartburn and regurgitation, which mainly occur after meals	[20,21]
Irritable bowel syndrome	Common functional gastrointestinal disorder characterized by the presence of chronic and recurrent abdominal discomfort	- Stress and psychological factors - Previous gastrointestinal infections and antibiotic use - Food and changes in the intestinal microbiota	Diarrhea, constipation, or an alternation between both	[22,23]
Inflammatory bowel disease	Encompasses a range of intestinal disorders characterized by a complex inflammatory response in the small and large intestines	History of ulcerative colitis and Crohn’s disease	Chronic inflammation of the gastrointestinal tract, abdominal pain, diarrhea, presence of blood in the stool, and weight loss	[24,25]
Gastrointestinal Cancer	It is a complex disease involving genetic and environmental factors, influenced by the host and its surroundings	- Infectious agents: *H. pylori*, hepatitis B and C virus, and human papillomavirus - Tobacco products - Diet, lack of physical activity, and/or energy balance disorder (obesity)	The luminal growth of tumors most of the time does not cause symptoms due to early-stage luminal obstruction	[26,27,28]

**Table 2:** Phytochemicals and the extraction methods of plants used for gastrointestinal disorders.

Scientific Name	Common Name	Part of the Plant	Associated Compounds	Extraction Method	References
*Nerium oleander*	Laurel rosa	Leaves	Oleandrin Digitoxingenin Urosolic acid	Infusion	[88]
*Dysphania* *Ambrosioides*	Epazote	Aerial parts	Syringic acid Quercetin Hesperetin Luteolin	Maceration	[89]
*Parthenium incanum*	Mariola	Leaves	Parthenin Coronopoline	Maceration	[90]
*Salvia officinalis*	Salvia	Leaves	Rosmarinic acid Salvianolic acid Catechin	Decoction	[91]
*Origanum majorana*	Mejorana	Aerial parts	Carvacrol Thymol Hydroquinone Arbutin	NA	[92]
*Ruta chalepensis*	Ruda	Leaves	Coumarins Hesperidin Acridine	Decoction	[93]
*Gnaphalium* *oxyphyllum*	Gordolobo	Stems and leaves	Chlorogenic acid Flavones	Maceration	[94]
*Tagetes erecta*	Cempazúchitl	Aerial parts	Dihydrotagetone Tagetones Terpinolene Piperitone	NA	[95]
*Origanum vulgare*	Orégano	Stems, leaves and flowers	Carvacrol Thymol Linalool *y*-Terpinene	NA	[96]
*Ocimum basilicum*	Albahaca	Leaves	Linalool Estragole Methyl eugenol	Hydrodistillation	[97]
*Piper auritum*	Hierba santa	Leaves	Safrol α-Terpinene	SCE	[98]
*Rosmarinus* *officinalis*	Romero	Leaves	1,8-cineole α-pinene Camphor	Hydrodistillation	[99]
*Aloysia citrodora*	Cedrón	Aerial parts	d,l-Limonene γ-Muurolene trans- chrysanthenyl acetate	Hydrodistillation	[100]
*Cymbopogon citratus*	Zacate limón	Leaves	L-linanool Limonene Furfide	Decoction	[101]
*Artemisia absinthium*	Ajenjo	Leaves	Artemisinin α-Thujone 4-Terpineo	NA	[102]

Extraction method: (SCE) supercritical extraction. (NA) not available.

## Data Availability

Not applicable.

## References

[1] Leader G., Murray M., O’súilleabháin P.S., Maher L., Naughton K., Arndt S., White K., Traina I., Mannion A. (2020). Relationship between parent-reported gastrointestinal symptoms, sleep problems, autism spectrum disorder symptoms, and behavior problems in children and adolescents with 22q11.2 deletion syndrome. Res. Dev. Disabil..

[2] Kang D.W., Adams J.B., Gregory A.C., Borody T., Chittick L., Fasano A., Khoruts A., Geis E., Maldonado J., McDonough-Means S. (2017). Microbiota Transfer Therapy alters gut ecosystem and improves gastrointestinal and autism symptoms: An open-label study. Microbiome.

[3] Liu J., Tan Y., Cheng H., Zhang D., Feng W., Peng C. (2022). Functions of Gut Microbiota Metabolites, Current Status and Future Perspectives. Aging Dis..

[4] Mazzocchi S., Visaggi P., Baroni L. (2023). Plant-based diets in gastrointestinal diseases: Which evidence?. Best Pract. Res. Clin. Gastroenterol..

[5] So D., Tuck C.J. (2021). Plant-based diets in gastrointestinal disorders: Something, nothing, or everything?. Lancet Gastroenterol. Hepatol..

[6] Sharma A., Flores-Vallejo R.d.C., Cardoso-Taketa A., Villarreal M.L. (2017). Antibacterial activities of medicinal plants used in Mexican traditional medicine. J. Ethnopharmacol..

[7] Sellami M., Slimeni O., Pokrywka A., Kuvačić G., Hayes L.D., Milic M., Padulo J. (2018). Herbal medicine for sports: A review. J. Int. Soc. Sports Nutr..

[8] Atanasov A.G., Waltenberger B., Pferschy-Wenzig E.M., Linder T., Wawrosch C., Uhrin P., Temml V., Wang L., Schwaiger S., Heiss E.H. (2015). Discovery and resupply of pharmacologically active plant-derived natural products: A review. Biotechnol Adv..

[9] Guldiken B., Ozkan G., Catalkaya G., Ceylan F.D., Yalcinkaya I.E., Capanoglu E. (2018). Phytochemicals of herbs and spices: Health versus toxicological effects. Food Chem. Toxicol..

[10] Feng W., Liu J., Ao H., Yue S., Peng C. (2020). Targeting gut microbiota for precision medicine: Focusing on the efficacy and toxicity of drugs. Theranostics.

[11] Contreras-Omaña R., Escorcia-Saucedo A.E., Velarde-Ruiz Velasco J.A. (2021). Prevalencia e impacto de resistencias a antimicrobianos en infecciones gastrointestinales: Una revisión. Rev. Gastroenterol. Mex..

[12] Sensoy I. (2021). A review on the food digestion in the digestive tract and the used in vitro models. Curr. Res. Food Sci..

[13] Ebrahimi P., Lante A. (2021). Polyphenols: A Comprehensive Review of their Nutritional Properties. Open Biotechnol. J..

[14] Wang R., Li Z., Liu S., Zhang D. (2023). Global, regional, and national burden of 10 digestive diseases in 204 countries and territories from 1990 to 2019. Front. Public Health.

[15] Peery A.F., Crockett S.D., Murphy C.C., Jensen E.T., Kim H.P., Egberg M.D., Lund J.L., Moon A.M., Pate V., Barnes E.L. (2022). Burden and Cost of Gastrointestinal, Liver, and Pancreatic Diseases in the United States: Update 2021. Gastroenterology.

[16] Yang H., Hu B. (2022). Immunological Perspective: Helicobacter pylori Infection and Gastritis. Mediators Inflamm..

[17] Li Y., Su Z., Li P., Li Y., Johnson N., Zhang Q., Du S., Zhao H., Li K., Zhang C. (2020). Association of Symptoms with Eating Habits and Food Preferences in Chronic Gastritis Patients: A Cross-Sectional Study. Evid.-Based Complement. Altern. Med..

[18] Wu Y., Murray G.K., Byrne E.M., Sidorenko J., Visscher P.M., Wray N.R. (2021). GWAS of peptic ulcer disease implicates *Helicobacter pylori* infection, other gastrointestinal disorders and depression. Nat. Commun..

[19] Serafim C., Araruna M.E., Alves Júnior E., Diniz M., Hiruma-Lima C., Batista L. (2020). A Review of the Role of Flavonoids in Peptic Ulcer (2010–2020). Molecules.

[20] Katz P.O., Dunbar K.B., Schnoll-Sussman F.H., Greer K.B., Yadlapati R., Spechler S.J. (2022). ACG Clinical Guideline for the Diagnosis and Management of Gastroesophageal Reflux Disease. Am. J. Gastroenterol..

[21] Delshad S.D., Almario C.V., Chey W.D., Spiegel B.M.R. (2019). Prevalence of Gastroesophageal Reflux Disease and Proton Pump Inhibitor-Refractory Symptoms. Gastroenterology.

[22] Chong P.P., Chin V.K., Looi C.Y., Wong W.F., Madhavan P., Yong V.C. (2019). The microbiome and irritable bowel syndrome—A review on the pathophysiology, current research and future therapy. Front. Microbiol..

[23] Pimentel M., Lembo A. (2020). Microbiome and Its Role in Irritable Bowel Syndrome. Dig. Dis. Sci..

[24] Stojanov S., Berlec A., Štrukelj B. (2020). The Influence of Probiotics on the Firmicutes/Bacteroidetes Ratio in the Treatment of Obesity and Inflammatory Bowel disease. Microorganisms.

[25] Guan Q.A. (2019). Comprehensive Review and Update on the Pathogenesis of Inflammatory Bowel Disease. J. Immunol. Res..

[26] Ağagündüz D., Cocozza E., Cemali Ö., Bayazıt A.D., Nanì M.F., Cerqua I., Morgillo F., Saygılı S.K., Berni Canani R., Amero P. (2023). Understanding the role of the gut microbiome in gastrointestinal cancer: A review. Front. Pharmacol..

[27] Waldum H., Fossmark R. (2021). Gastritis, Gastric Polyps and Gastric Cancer. Int. J. Mol. Sci..

[28] Sung H., Ferlay J., Siegel R.L., Laversanne M., Soerjomataram I., Jemal A., Bray F. (2021). Global Cancer Statistics 2020: GLOBOCAN Estimates of Incidence and Mortality Worldwide for 36 Cancers in 185 Countries. CA Cancer J. Clin..

[29] Paone P., Cani P.D. (2020). Mucus barrier, mucins and gut microbiota: The expected slimy partners?. Gut.

[30] Thursby E., Juge N. (2017). Introduction to the human gut microbiota. Biochem. J..

[31] Rinninella E., Raoul P., Cintoni M., Franceschi F., Miggiano G.A.D., Gasbarrini A., Mele M.C. (2019). What is the Healthy Gut Microbiota Composition? A Changing Ecosystem across Age, Environment, Diet, and Diseases. Microorganisms.

[32] Wallig M.A., Wallig M.A., Bolon B., Haschek W.M., Rousseaux C.G. (2018). Digestive System. Fundamentals of Toxicologic Pathology.

[33] Mirzaei R., Dehkhodaie E., Bouzari B., Rahimi M., Gholestani A., Hosseini-Fard S.R., Keyvani H., Teimoori A., Karampoor S. (2022). Dual role of microbiota-derived short-chain fatty acids on host and pathogen. Biomed. Pharmacother..

[34] Wilson A.S., Koller K.R., Ramaboli M.C., Nesengani L.T., Ocvirk S., Chen C., Flanagan C.A., Sapp F.R., Merritt Z.T., Bhatti F. (2020). Diet and the Human Gut Microbiome: An International Review. Dig. Dis. Sci..

[35] Valdes A.M., Walter J., Segal E., Spector T.D. (2018). Role of the gut microbiota in nutrition and health. BMJ.

[36] Labanski A., Langhorst J., Engler H., Elsenbruch S. (2020). Stress and the brain-gut axis in functional and chronic-inflammatory gastrointestinal diseases: A transdisciplinary challenge. Psychoneuroendocrinology.

[37] Sasso J.M., Ammar R.M., Tenchov R., Lemmel S., Kelber O., Grieswelle M., Zhou Q.A. (2023). Gut Microbiome-Brain Alliance: A Landscape View into Mental and Gastrointestinal Health and Disorders. ACS Chem. Neurosci..

[38] Durack J., Lynch S.V. (2018). The gut microbiome: Relationships with disease and opportunities for therapy. J. Exp. Med..

[39] Katzung B.G. (2017). Basic and Clinical Pharmacology.

[40] Jamshidi-Kia F., Lorigooini Z., Amini-Khoei H. (2018). Medicinal plants: Past history and future perspective. J. Herbmed Pharmaco..

[41] Marrelli M. (2021). Medicinal Plants. Plants.

[42] Van Wyk A.S., Prinsloo G. (2018). Medicinal plant harvesting, sustainability and cultivation in South Africa. Biol. Conserv..

[43] He J., Yang B., Dong M., Wang Y. (2018). Crossing the roof of the world: Trade in medicinal plants from Nepal to China. J. Ethnopharmacol..

[44] Roy A., Jauhari N., Bharadvaja N., Akhtar M.S., Swamy M.K. (2018). Medicinal plants as a potential source of chemopreventive agents. Anticancer Plants: Natural Products and Biotechnological Implements.

[45] Torres-León C., Rebolledo Ramírez F., Aguirre-Joya J.A., Ramírez-Moreno A., Chávez-González M.L., Aguillón-Gutierrez D.R., Camacho-Guerra L., Ramírez-Guzmán N., Hernández Vélez S., Aguilar C.N. (2023). Medicinal plants used by rural communities in the arid zone of Viesca and Parras Coahuila in northeast Mexico. Saudi Pharm. J..

[46] Astutik S., Pretzsch J., Kimengsi J.N. (2019). Asian Medicinal Plants’ Production and Utilization Potentials: A Review. Sustainability.

[47] Spina D., Barbieri C., Carbone R., Hamam M., D’Amico M., Di Vita G. (2023). Market Trends of Medicinal and Aromatic Plants in Italy: Future Scenarios Based on the Delphi Method. Agronomy.

[48] Oguntibeju O.O. (2018). Medicinal plants with anti-inflammatory activities from selected countries and regions of Africa. J. Inflamm. Res..

[49] Mata R., Figueroa M., Navarrete A., Rivero-Cruz I. (2019). Chemistry and Biology of Selected Mexican Medicinal Plants. Prog. Chem. Org. Nat. Prod..

[50] Guzmán Maldonado H., Díaz Huacuz R., González Chavira M. (2017). Plantas Medicinales la Realidad de una Tradición Ancestral. https://vun.inifap.gob.mx/VUN_MEDIA/BibliotecaWeb/_media/_folletoinformativo/1044_4729_Plantas_medicinales_la_realidad_de_una_tradici%C3%B3n_ancestral.pdf.

[51] Gould C.V., Free R.J., Bhatnagar J., Soto R.A., Royer T.L., Maley W.R., Moss S., Berk M.A., Craig-Shapiro R., Kodiyanplakkal R.P.L. (2023). Transmission of yellow fever vaccine virus through blood transfusion and organ transplantation in the USA in 2021: Report of an investigation. Lancet Microbe.

[52] Ogunyemi S.O., Zhang F., Abdallah Y., Zhang M., Wang Y., Sun G., Qiu W., Li B. (2019). Biosynthesis and characterization of magnesium oxide and manganese dioxide nanoparticles using *Matricaria chamomilla* L. extract and its inhibitory effect on *Acidovorax oryzae* strain RS-2. Artif. Cells Nanomed. Biotechnol..

[53] Akram W., Ahmed S., Rihan M., Arora S., Khalid M., Ahmad S., Ahmad F., Haque S., Vashishth R. (2024). An updated comprehensive review of the therapeutic properties of Chamomile (*Matricaria chamomilla* L.). Int. J. Food Prop..

[54] El Joumaa M.M., Borjac J.M. (2022). *Matricaria chamomilla*: A valuable insight into recent advances in medicinal uses and pharmacological activities. Phytochem. Rev..

[55] El Mihyaoui A., Esteves da Silva J.C.G., Charfi S., Candela Castillo M.E., Lamarti A., Arnao M.B. (2022). Chamomile (*Matricaria chamomilla* L.): A Review of Ethnomedicinal Use, Phytochemistry and Pharmacological Uses. Life.

[56] Chrysargyris A., Papakyriakou E., Petropoulos S.A., Tzortzakis N. (2019). The combined and single effect of salinity and copper stress on growth and quality of *Mentha spicata* plants. J. Hazard. Mater..

[57] Mahboubi M. (2021). *Mentha spicata* L. essential oil, phytochemistry and its effectiveness in flatulence. J. Tradit. Med. Complement..

[58] el Menyiy N., Mrabti H.N., el Omari N., Bakili A.E., Bakrim S., Mekkaoui M., Balahbib A., Amiri-Ardekani E., Ullah R., Alqahtani A.S. (2022). Medicinal Uses, Phytochemistry, Pharmacology, and Toxicology of *Mentha spicata*. Evid.-Based Complement. Altern. Med..

[59] Mahendran G., Verma S.K., Rahman L.U. (2021). The traditional uses, phytochemistry and pharmacology of spearmint (*Mentha spicata* L.): A review. J. Ethnopharmacol..

[60] Saqib S., Ullah F., Naeem M., Younas M., Ayaz A., Ali S., Zaman W. (2022). Mentha: Nutritional and Health Attributes to Treat Various Ailments Including Cardiovascular Diseases. Molecules.

[61] Petrisor G., Motelica L., Craciun L.N., Oprea O.C., Ficai D., Ficai A. (2022). *Melissa officinalis*: Composition, Pharmacological Effects and Derived Release Systems—A Review. Int. J. Mol. Sci..

[62] Stoyanova N., Spasova M., Manolova N., Rashkov I., Kamenova-Nacheva M., Staleva P., Tavlinova-Kirilova M. (2023). Electrospun PLA-Based Biomaterials Loaded with *Melissa officinalis* Extract with Strong Antioxidant Activity. Polymers.

[63] Ghazizadeh J., Hamedeyazdan S., Torbati M., Farajdokht F., Fakhari A., Mahmoudi J., Araj-khodaei M., Sadigh-Eteghad S. (2020). *Melissa officinalis* L. hydro-alcoholic extract inhibits anxiety and depression through prevention of central oxidative stress and apoptosis. Exp. Physiol..

[64] Miraj S., Rafieian-Kopaei M., Kiani S. (2017). *Melissa officinalis* L: A Review Study With an Antioxidant Prospective. Evid. Based Complement. Alternat Med..

[65] Swor K., Poudel A., Satyal P., Setzer W.N. (2024). The Essential Oil Compositions of *Ambrosia acanthicarpa* Hook., *Artemisia ludoviciana* Nutt., and *Gutierrezia sarothrae* (Pursh) Britton & Rusby (Asteraceae) from the Owyhee Mountains of Idaho. Molecules.

[66] Kamarauskaite J., Baniene R., Raudone L., Vilkickyte G., Vainoriene R., Motiekaityte V., Trumbeckaite S. (2021). Antioxidant and mitochondria-targeted activity of caffeoylquinic-acid-rich fractions of wormwood *(Artemisia absinthium* L.) and silver wormwood (*Artemisia ludoviciana* Nutt.). Antioxidants.

[67] Palacios-Espinosa J.F., Núñez-Aragón P.N., Gomez-Chang E., Linares E., Bye R., Romero I. (2021). Anti-Helicobacter pylori Activity of *Artemisia ludoviciana* subsp. mexicana and Two of Its Bioactive Components, Estafiatin and Eupatilin. Molecules.

[68] Ezeta-Miranda A., Vera-Montenegro Y., Avila-Acevedo J.G., García-Bores A.M., Estrella-Parra E.A., Francisco-Marquez G., Ibarra-Velarde F. (2020). Efficacy of purified fractions of *Artemisia ludoviciana* Nutt. mexicana and ultraestructural damage to newly excysted juveniles of Fasciola hepatica in vitro. Vet. Parasitol..

[69] Rivero-Cruz I., Anaya-Eugenio G., Pérez-Vásquez A., Martínez A.L., Mata R. (2017). Quantitative Analysis and Pharmacological Effects of *Artemisia ludoviciana* Aqueous Extract and Compounds. Nat. Prod. Commun..

[70] Jasso de Rodríguez D., Puente-Romero G.N., Díaz-Jiménez L., Rodríguez-García R., Ramírez-Rodríguez H., Villarreal-Quintanilla J.A., Flores-López M.L., Carrillo-Lomelí D.A., Genisheva Z.A. (2019). In vitro gastrointestinal digestion of microencapsulated extracts of *Flourensia cernua, F. microphylla*, and *F. retinophylla*. Ind. Crops Prod..

[71] Jasso de Rodríguez D., Salas-Méndez E.d.J., Rodríguez-García R., Hernández-Castillo F.D., Díaz-Jiménez M.L.V., Sáenz-Galindo A., González-Morales S., Flores-López M.L., Villarreal-Quintanilla J.A., Peña-Ramos F.M. (2017). Antifungal activity in vitro of ethanol and aqueous extracts of leaves and branches of *Flourensia* spp. against postharvest fungi. Ind. Crops Prod..

[72] Alvarez-Pérez O.B., Ventura-Sobrevilla J.M., Ascacio-Valdés J.A., Rojas R., Verma D.K., Aguilar C.N. (2020). Valorization of *Flourensia cernua* DC as source of antioxidants and antifungal bioactives. Ind. Crops Prod..

[73] Assanga S.B.I., Luján L.M.L., Ruiz J.C.G., McCarty M.F., Cota-Arce J.M., Espinoza C.L.L., Salido A.A.G., Ángulo D.F. (2020). Comparative analysis of phenolic content and antioxidant power between parasitic *Phoradendron californicum* (toji) and their hosts from Sonoran Desert. Results Chem..

[74] Iloki-Assanga S.B., Lewis-Luján L.M., Lara-Espinoza C.L., Gil-Salido A.A., Fernandez-Angulo D., Rubio-Pino J.L., Haines D.D. (2015). Solvent effects on phytochemical constituent profiles and antioxidant activities, using four different extraction formulations for analysis of *Bucida buceras* L. and *Phoradendron californicum* Complementary and Alternative Medicine. BMC Res. Notes.

[75] Mathiasen R.L. (2016). The classification of California Viscaceae: An alternative perspective. Madroño.

[76] Martínez-Ávila G.C.G., Aguilar-Zarate P., Rojas R. (2021). Currently Applied Extraction Processes for Secondary Metabolites from *Lippia turbinata* and *Turnera diffusa* and Future Perspectives. Separations.

[77] Chaurasiya N.D., Zhao J., Pandey P., Doerksen R.J., Muhammad I., Tekwani B.L. (2019). Selective Inhibition of Human Monoamine Oxidase B by Acacetin 7-Methyl Ether Isolated from *Turnera diffusa* (Damiana). Molecules.

[78] Tousson E., Hafez E., Zaki S., Gad A., Elgharabawy R.M. (2020). Evaluation of the testicular protection conferred by damiana (*Turnera diffusa Willd*.) against amitriptyline-induced testicular toxicity, DNA damage and apoptosis in rats. Biomed. Pharmacother..

[79] Szewczyk K., Zidorn C. (2014). Ethnobotany, phytochemistry, and bioactivity of the genus *Turnera (Passifloraceae*) with a focus on damiana—*Turnera diffusa*. J. Ethnopharmacol..

[80] Urbizu-González A.L., Castillo-Ruiz O., Martínez-Ávila G.C.G., Torres-Castillo J.A. (2017). Natural variability of essential oil and antioxidants in the medicinal plant *Turnera diffusa*. Asian Pac. J. Trop. Med..

[81] Yuan H., Ma Q., Ye L., Piao G. (2016). The Traditional Medicine and Modern Medicine from Natural Products. Molecules.

[82] Batiha G.E.S., Alkazmi L.M., Wasef L.G., Beshbishy A.M., Nadwa E.H., Rashwan E.K. (2020). *Syzygium aromaticum* L. (*Myrtaceae*): Traditional Uses, Bioactive Chemical Constituents, Pharmacological and Toxicological Activities. Biomolecules.

[83] Feng W., Ao H., Peng C. (2018). Gut microbiota, short-chain fatty acids, and herbal medicines. Front. Pharmacol..

[84] An X., Bao Q., Di S., Zhao Y., Zhao S., Zhang H., Lian F., Tong X. (2019). The interaction between the gut Microbiota and herbal medicines. Biomed. Pharmacother..

[85] Gasaly N., Riveros K., Gotteland M. (2020). Fitoquímicos: Una nueva clase de prebióticos. Rev. Chil. Nutr..

[86] Hussein R.A., A. El-Anssary A.A., Builders P.F. (2019). Plants Secondary Metabolites: The Key Drivers of the Pharmacological Actions of Medicinal Plants. Herbal Medicine.

[87] Li Y., Kong D., Fu Y., Sussman M.R., Wu H. (2020). The effect of developmental and environmental factors on secondary metabolites in medicinal plants. Plant. Physiol. Biochem..

[88] Kanwal N., Rasul A., Hussain G., Anwar H., Shah M.A., Sarfraz I., Riaz A., Batool R., Shahbaz M., Hussain A. (2020). Oleandrin: A bioactive phytochemical and potential cancer killer via multiple cellular signaling pathways. Food Chem. Toxicol..

[89] Kandsi F., Conte R., Marghich M., Lafdil F.Z., Alajmi M.F., Bouhrim M., Mechchate H., Hano C., Aziz M., Gseyra N. (2021). Phytochemical Analysis, Antispasmodic, Myorelaxant, and Antioxidant Effect of *Dysphania ambrosioides* (L.) Mosyakin and Clemants Flower Hydroethanolic Extracts and Its Chloroform and Ethyl Acetate Fractions. Molecules.

[90] Hernández-Marín D.A., Castro-Rios R., Chávez-Montes A., Castillo-Hernández S.L., Elizondo-Luevano J.H., Muñoz-Ortega M.H., Sánchez-García E. (2024). Antiparasitic Activity of Isolated Fractions from *Parthenium incanum* Kunth against the Hemoflagellate Protozoan *Trypanosoma cruzi*. Antibiotics.

[91] Jedidi S., Sammari H., Selmi H., Hosni K., Rtibi K., Aloui F., Adouni O., Sebai H. (2021). Strong protective effects of *Salvia officinalis* L. leaves decoction extract against acetic acid-induced ulcerative colitis and metabolic disorders in rat. J. Funct. Foods.

[92] Bouyahya A., Chamkhi I., Benali T., Guaouguaou F.E., Balahbib A., El Omari N., Taha D., Belmehdi O., Ghokhan Z., el Menyiy N. (2021). Traditional use, phytochemistry, toxicology, and pharmacology of *Origanum majorana* L.. J. Ethnopharmacol..

[93] Khadhri A., Bouali I., Belkhir S., Mokded R., Smiti S., Falé P., Araújo M.E.M., Serralheiro M.L.M. (2017). In vitro digestion, antioxidant and antiacetylcholinesterase activities of two species of *Ruta: Ruta chalepensis* and *Ruta montana*. Pharm. Biol..

[94] Heredia-Castro P.Y., García-Baldenegro C.V., Santos-Espinosa A., Tolano-Villaverde I.d.J., Manzanarez-Quin C.G., Valdez-Domínguez R.D., Ibarra-Zazueta C., Osuna-Chávez R.F., Rueda-Puente E.O., Hernández-Moreno C.G. (2022). Perfil fitoquímico, actividad antimicrobiana y antioxidante de extractos de *Gnaphalium oxyphyllum* y *Euphorbia maculata* nativas de Sonora. Rev. Mex. Cienc. Pecu..

[95] Salehi B., Valussi M., Flaviana Bezerra Morais-Braga M., Nalyda Pereira Carneiro J., Linkoln Alves Borges Leal A., Douglas Melo Coutinho H., Vitalini S., Kręgiel D., Antolak H., Sharifi-Rad M. (2018). *Tagetes* spp. Essential Oils and Other Extracts: Chemical Characterization and Biological Activity. Molecules.

[96] Lombrea A., Antal D., Ardelean F., Avram S., Pavel I.Z., Vlaia L., Mut A.M., Diaconeasa Z., Dehelean C.A., Soica C. (2020). A Recent Insight Regarding the Phytochemistry and Bioactivity of *Origanum vulgare* L.. Essential Oil. Int. J. Mol. Sci..

[97] Purushothaman B., Prasannasrinivasan R., Suganthi P., Ranganathan B., Gimbun J., Shanmugam K. (2018). A Comprehensive Review on *Ocimum basilicum*. J. Nat. Remedies..

[98] Conde-Hernández L.A., Espinosa-Victoria J.R., Guerrero-Beltrán J. (2017). Supercritical extraction of essential oils of *Piper auritum* and *Porophyllum ruderale*. J. Supercrit. Fluids.

[99] Satyal P., Jones T.H., Lopez E.M., McFeeters R.L., Ali N.A.A., Mansi I., Al-Kaf A.G., Setzer W.N. (2017). Chemotypic Characterization and Biological Activity of *Rosmarinus officinalis*. Foods.

[100] Rashid H.M., Mahmod A.I., Afifi F.U., Talib W.H. (2022). Antioxidant and Antiproliferation Activities of Lemon Verbena (*Aloysia citrodora):* An In Vitro and In Vivo Study. Plants.

[101] Oladeji O.S., Adelowo F.E., Ayodele D.T., Odelade K.A. (2019). Phytochemistry and pharmacological activities of *Cymbopogon citratus*: A review. Sci. Afr..

[102] Batiha G.E.S., Olatunde A., El-mleeh A., Hetta H.F., Al-rejaie S., Alghamdi S., Zahoor M., Beshbishy A.M., Murata T., Zaragoza-bastida A. (2020). Bioactive Compounds, Pharmacological Actions, and Pharmacokinetics of Wormwood (*Artemisia absinthium*). Antibiotics.

[103] Wang W., Xu J., Fang H., Li Z., Li M. (2020). Advances and challenges in medicinal plant breeding. Plant Sci..

[104] Süntar I. (2020). Importance of ethnopharmacological studies in drug discovery: Role of medicinal plants. Phytochem. Rev..

[105] Joshi V.K., Joshi A., Dhiman K.S. (2017). The Ayurvedic Pharmacopoeia of India, development and perspectives. J. Ethnopharmacol..

[106] Schifter Aceves L. (2014). Las Farmacopeas Mexicanas en la construcción de la identidad nacional. Rev. Mex. Cienc. Farm..

[107] Iannitti T., Morales-Medina J.C., Bellavite P., Rottigni V., Palmieri B. (2016). Effectiveness and Safety of *Arnica Montana* in Post-Surgical Setting, Pain and Inflammation. Am. J. Ther..

[108] Sotiropoulou N.S., Megremi S.F., Tarantilis P. (2020). Evaluation of antioxidant activity, toxicity, and phenolic profile of aqueous extracts of chamomile (*Matricaria chamomilla* L.) and sage (*Salvia officinalis* L.) prepared at different temperatures. Appl. Sci..

[109] Yang X., Han H., Li B., Zhang D., Zhang Z., Xie Y. (2021). Fumigant toxicity and physiological effects of spearmint (*Mentha spicata*, Lamiaceae) essential oil and its major constituents against Reticulitermes dabieshanensis. Ind Crop Prod..

[110] Stojanović N.M., Randjelović P.J., Mladenović M.Z., Ilić I.R., Petrović V., Stojiljković N., Ilić S., Radulović N.S. (2019). Toxic essential oils, part VI: Acute oral toxicity of lemon balm (*Melissa officinalis* L.) essential oil in BALB/c mice. Food Chem. Toxicol..

[111] Gálvez Romero J.L., Parada Sosa C.M., Burgoa G.L., Lorenzo Leal A.C., El Kassis E.G., Bautista Rodríguez E., Paredes Juárez G.A., Hernández L.R., Bach H., Juárez Z.N. (2022). Antimycobacterial, cytotoxic, and anti-inflammatory activities of *Artemisia ludoviciana*. J. Ethnopharmacol..

[112] Jasso de Rodríguez D., Torres-Moreno H., López-Romero J.C., Vidal-Gutiérrez M., Villarreal-Quintanilla J.A., Carrillo-Lomelí D.A., Robles-Zepeda R.E., Vilegas W. (2023). Antioxidant, Anti-Inflammatory, and Antiproliferative Activities of *Flourensia* spp.. Biocatal. Agric. Biotechnol..

[113] Mendez-Pfeiffer P., Ballesteros-Monrreal M.G., Leyva M., Ortega-Garcia J., Montaño-Leyva B., Valencia D., Aguilar-Martinez M. (2024). Actividad Antioxidante, Antiproliferativa y Antibacteriana de Extractos de *Phoradendron californicum*; una Planta Parásita del Noroeste de México. Biotecnia.

[114] El-Demerdash F.M., Jebur A.B., Nasr H.M., Hamid H.M. (2019). Modulatory effect of *Turnera diffusa* against testicular toxicity induced by fenitrothion and/or hexavalent chromium in rats. Environ. Toxicol..

